# Emergent hybrid surgical approaches for non-dissecting ruptured Kommerell's aneurysm: a case report series

**DOI:** 10.1186/s13019-023-02156-x

**Published:** 2023-03-24

**Authors:** Alejandro Velandia-Sánchez, Sebastián Gómez-Galán, Sebastian Gallo-Bernal, Camilo A. Polania-Sandoval, Ivonne G. Pineda-Rodríguez, Paula Florez-Amaya, Lina M. Sanabria-Arévalo, Julián Senosiain-González, Juan G. Barrera-Carvajal, Juan P. Umana, Jaime Camacho-Mackenzie

**Affiliations:** 1 Department of Cardiovascular Surgery, Fundación Cardioinfantil-Instituto de Cardiología, Bogotá, Colombia; 2 Vascular and Endovascular Surgery Research Group, Fundación Cardioinfantil-Instituto de Cardiología, Cra 13B No. 161-85 Torre I Piso 8, 110131 Bogotá, Colombia; 3 School of Medicine and Health Sciences, Universidad del Rosario, Bogotá, Colombia; 4 Division of Radiology, Massachusetts General Hospital, Boston, MA USA

**Keywords:** Kommerell’s aneurysm, Frozen elephant trunk, Hybrid procedures, Surgical approach

## Abstract

**Background:**

Kommerell’s aneurysm is a saccular or fusiform dilatation found in 3–8% of Kommerell’s diverticulum cases. A non-dissecting rupture rate of 6% has been reported. If ruptured, emergent surgical correction is usually granted. However, evidence regarding the optimal surgical approach in this acute setting is scarce. In this case report series, we aim to describe our experience managing type-1 non-dissecting ruptured Kommerell's aneurysm with hybrid emergent surgical approaches.

**Cases presentation:**

From January 2005 to December 2020, three cases of type-1 non-dissecting ruptured Kommerell's aneurysm requiring emergent surgical repair were identified. The mean age was 66.67 ± 7.76 years, and 3/3 were male. The most common symptoms were atypical chest pain, dyspnoea, and headache (2/3). The mean aneurysm’s diameter was 63.67 ± 5.69 mm. Frozen Elephant Trunk was the preferred surgical approach (2/3). The Non-Frozen Elephant Trunk patient underwent a hybrid procedure consisting of a supra-aortic debranching and a zone-2 stent-graft deployment. We found a mean clamp time of 140 ± 60.75 min, cardiac arrest time of 51.33 ± 3.06 min, and a hospital stay of 13.67 ± 5.51 days. The most common complications were surgical-site infection and shock (2/3). Only one patient died (1/3).

**Conclusion:**

Evidence of management for non-dissecting ruptured Kommerell's aneurysms is scarce. Additional, robust, and more extensive studies are required. The selection of the appropriate surgical approach is challenging, and each patient should be individualized. Frozen Elephant Trunk was feasible for patients requiring emergent surgical repair in our centre. However, other hybrid or open procedures can be performed.

## Background

Kommerell's Diverticulum (KD) is a rare developmental abnormality of the aorta, associated in 20–60% of the cases with an aberrant subclavian artery (AScA) [1, 2]. It is currently known that KD results from an abnormal embryological remanent, secondary to a failed regression of the fourth primitive aortic arch [2]. According to its pathogenesis, KD can be classified into three different types: Type I KD is characterised by a KD secondary to an aberrant right subclavian artery (aRScA) in a left aortic arch; Type II KD is derived from an aberrant left subclavian artery (aLScA) with a concomitant right aortic arch; and finally, Type III KD is a diverticulum arising from the isthmus of the thoracic aorta without the presence of an ASCA [1, 3, 4]. 

A Kommerell's Aneurysm (KA) is defined as a fusiform or saccular (most frequent) dilatation of a KD, with a reported average size of 50.7 ± 7.1 mm [5]. They are extremely rare, as they can be found only in 3–8% of the KD [5–7]. Some authors have stated that a KA should be defined when the size of a KD is greater than 30 mm, since the risk of rupture (rKA) increases significantly, yet there is still no consensus [8, 9]. Histological studies highlight that KA requires a separate categorisation that distinguishes them from KD, as it has been demonstrated that medial cystic necrosis is present in the vessel wall of the KA [10]. It is hypothesised that this structural alteration relates to a higher frequency of fatal complications, such as distal embolisation, compression of adjacent structures, dissection, and rupture [7, 10]. 

KA tends to be asymptomatic. However, when symptoms are present, they could be attributed to complications. This is the case of *dysphagia lusoria*, a classic sign of an AScA, described as an impairment of swallowing due to oesophagal compression from an aberrant artery passing posterior to the oesophagus [1, 5]. Similarly, when the KA presents a dissection or rupture, the clinical presentation becomes alarming with signs or symptoms like rest dyspnoea, lancinating chest pain, or hemodynamic instability [2]. 

It has been described that KA has a concomitant dissection and rupture rate between 19 and 53% and a non-dissecting rupture rate range of 6–19% [2, 6]. Due to the implications of this condition, including death, emergent surgical repair is usually granted. Some elective surgical approaches have been proposed; however, evidence regarding the optimal emergent management for KA and rKA is mainly based on isolated case reports or a few KD cohorts, as shown in Table 1 [2, 5–22].

**Table 1:** Summary of reported Komerell's aneurysms reported in the literature

Authors	Refs	Study title	Study design	Patients involved	Patient age (years)	Clinical presentation	KA type	KA diameter (mm)*	Surgical approach	Surgical technique performed
Cinà et al	[8]	Kommerell's diverticulum and right-sided aortic arch: a cohort study and review of the literature	Case series	3	Mean 47 (Range 21–67)	1/3 claudication of the left arm. 1/3 dysphagia, epigastric discomfort, and palpitations. 1/3 dry cough and occasional dysphagia	Type II	Mean 41 (range 30–64)	Open approach	3/3 A left subclavian-carotid transposition was performed. 2/3 interposition graft. 1/3 endoaneurysmorrhaphy
Kaki et al	[11]	Kommerell's Diverticular Rupture Complicated by Aberrant Left Subclavian Artery and Right Aortic Arch Successfully Treated Surgically	Case report	1	73	Chest and back pain	Ruptured Type II	35	Open approach	Initial open proximal anastomosis was performed with a branched vascular graft. Then the vascular graft was clamped, and reperfusion of the upper part of the body was started for distal anastomosis site of the KA after reconstruction of the aLScA
Kuan-Ming et al	[37]	Kommerell’s aneurysm	Case report	1	54	Sudden onset chest pain with radiation to back	Non-Ruptured dissecting Type II	Non-reported	Open approach	Thoracic aortic replacement from mid-arch to low thoracic aorta and reconstruction of both subclavian arteries
Kouchoukos et al	[7]	Aberrant subclavian artery and Kommerell aneurysm: Surgical treatment with a standard approach	Case series	10	Mean 65 (range 46–82)	6/10 presented chest, back, or shoulder pain. 1/10 had paralysis of the left vocal cord. 3/10 were asymptomatic	8/10 Type I 2/10 Type II	Mean 58 (range 35–77)	Open approach	4/10 underwent preliminary carotid-to-aberrant subclavian artery bypass and ligation of the subclavian artery proximal to the origin of the vertebral artery. 10/10 had graft replacement of the subclavian artery and descending thoracic aneurysms. 6/10 ontinuity of the aberrant subclavian artery was preserved
Cruz-Siria et al	[13]	Hybrid treatment of a symptomatic aneurysm of a Kommerell's diverticulum	Case report	1	62	Chest pain and dysphagia	Type I	32	Two-staged hybrid approach	Left carotid-subclavian bypass. After two months TEVAR + arteriotomy of right subclavian artery + coil embolizationn + posterior right subclavian artery reimplantation at the origin of right carotid artery
Yamashiro et al	[22]	Endovascular repair of intrathoracic ruptured Kommerell’s diverticulum	Case report	1	55	Sudden onset of excruciating chest pain and syncope with hypotension	Type II	Non-reported	One-stage hybrid approach	Endovascular stent-graft repair with total open debranching of the neck vessels
Lamb et al	[14]	Hybrid endovascular treatment of an aberrant right subclavian artery with Kommerell aneurysm	Case report	1	82	Dishagia lussoria, weight loss and chest pain	Type I	35	Tree-staged hybrid approach	Left carotid-subclavian bypass, right carotid-subclavian bypass and TEVAR
Lococo et al	[9]	Successful Conservative Treatment of a Kommerell Aneurysm Associated With Right-Sided Aortic Arch	Case report	1	73	Incidental finding, asymptomatic	Type II	29	Conservative approach	Radiologic surveillance due to advanced malignancy
Ben Abdallah et al	[15]	Random Finding of a Ruptured Kommerell Aneurysm After Stroke	Case report	1	74	Worsening of disphagia and chest pain, incidental finding	Ruptured Type I	81	One-stage hybrid approach	TEVAR + cervical debranching of the aLScA, then left carotid endarterectomy
Wong et al	[5]	Saccular Kommerell aneurysm, a potential pitfall on MDCT imaging – A review of imaging features and potential mimics	Case report	1	78	Chest pain and anterolateral ST elevation	Saccular Type I	41	Non-reported	Surgical approach, no details
Tzilalis et al	[16]	Hybrid treatment for a type 2 Kommerell’s aneurysm in a nonagenarian	Case report	1	90	Synchronous diaphragmatic flutter	Type II	117	One-stage hybrid approach	Left common carotid artery-subclavian bypass and thoracic endografting distal to the origin of the RSA. A vascular plug occluded the aLScA
Madjarov et al	[21]	Single-stage hybrid repair of a ruptured Kommerell diverticulum associated with dextrorotation, bovine arch, and bicuspid aortic valve	Case report	1	44	Severe mid-sternal chest pain radiating to the back	Type III	85	One-stage hybrid approach	Subclavian artery revascularization, aortic resection with open proximal anastomosis, TEVAR, and valve repair
Ikeno et al	[2]	Graft Replacement of Kommerell Diverticulum and In Situ Aberrant Subclavian Artery Reconstruction	Case series	17	Mean 68.2 ± 12.7 (range 42–89)	11/17 asymptomatic. 2/17 dysphagia. 2/17 chest pain. 1/17 chest discomfort. 1/17 hoarseness	6/17 Type I 1/6 Type I rKA dissecting not-ruptured 1/6 Type I rKA non-dissceting. 11/17 Type II 1/11 type II rKA	Mean 50.1 ± 7.3 (range 32.9–66)	Open approach	5/17 Total arch replacement through a median sternotomy. 9/17 Graft replacement of the descending aorta through a posterolateral thoracotomy. 2/17 Extensive replacement from the aortic arch to the descending aorta through a posterolateral thoracotomy
Sica et al	[20]	First MDCT evidence of ruptured aberrant left subclavian artery aneurysm in right aortic arch, Kommerell’s diverticulum and extrapleural hematoma treated by emergency thoracic endovascular aortic repair	Case report	1	74	6 h of abrupt onset of acute chest pain, anemia, hypotension, and shortness of breath	Saccular Ruptured non-dissecting Type II	70	Two-staged hybrid approach	Emergency TEVAR and an Amplatzer vascular plug was placed into the first segment of the aLScA. 20 h later a left carotid-axillary surgical bypass
Singh et al	[17]	Intraoperative retrograde TEVAR to control endoleak after emergent total arch replacement and frozen elephant trunk repair for ruptured Kommerell's diverticulum	Case report	1	62	Acute onset tearing chest pain	Dissecting Ruptured Type I + Intramural Hematoma	40	One-stage hybrid approach	Emergent total arch replacement + FET
Mattioli et al	[10]	Unusual cause of back pain and dysphagia: a Kommerell aneurysm	Case report	1	85	Back pain and dysphagia that started	Saccular Type I	65	Conservative approach	Conservative, no details
Herrán de la Gala et al	[18]	Kommerell diverticulum aneurysm and bicuspid aortic valve	Case report	1	54	Incidental finding, follow-up by cardiology due to a bicuspid aortic valve and moderate stable angina with progressive worsening	Saccular Type I	66	One-stage hybrid approach	Double coronary artery bypass + FET
Gergen et al	[19]	Endovascular aortic repair of Kommerell diverticulum associated with aberrant left subclavian artery	Case report	1	62	Shortness of breath, dysphagia, and an unintentional 20-pound weight loss over the last three months	Type I	35	Tree-staged hybrid approach	Left carotid-subclavian bypass, TEVAR, and finally VATS division of the aberrant left subclavian artery and vascular ring
Godfrin et al	[6]	Kommerell Aneurysm	Case report	1	73	Hemoptysis due to lung mass, incidental finding	Non-reported	Non-reported	Conservative approach	Radiologic surveillance due to malignancy

*KA were considered to be those larger than 30 mm, even though they were described as KD

This article aims to describe the clinical characteristics and hybrid emergent surgical approaches for three patients with type-1 non-dissecting rKA in a single referral centre for cardiovascular surgery in Latin America.

## Cases presentation

We present three patients diagnosed with type-1 non-dissecting rKA who underwent emergent hybrid surgical repair. Cases are summarised in Table 2.

**Table 2:** Characteristics of patients with type-1 non-dissecting rKA managed with an emergent hybrid approach presented in this article

**Patients characteristics**		
**Clinical presentation**		
Sociodemographic information	Mean age (years)	66.67 ± 7.76
	Male	3/3
	Hypertension	2/3
Symptoms	Dyspnea	2/3
	Dysfagia	1/3
	Dysphonia	1/3
	Atypical chest pain	2/3
	General malaise	1/3
	Lipothymia	1/3
	Headache	2/3
Signs	Hypotension	2/3
	Tachycardia	2/3
	Tachypnea	2/3
**CT Findings**		
KA Characteristics	Mean diameter (mm)	63.67 ± 5.69
	Ruptured KA	2/3
	Unruptured KA	1/3
**Emergent surgical approach**		
Hybrid Procedure Performed	Frozen Elephant Trunk	2/3
	Carotid-to subclavian bypass + TEVAR	1/3
Operative times	Mean Clamp Time (min)	140 ± 60.75
	Mean Cardiac Arrest Time (min)	51.33 ± 3.06
**Postoperative data**		
Mean Hospital Stay Time		13.67 ± 5.51
Medical complications	Infection	2/3
	Shock	2/3
	Others	2/3
	Death	1/3

### Case number 1 (frozen elephant trunk (FET))

A 73-year-old male presented to the emergency room (ER) with intense pain in the left thoracoabdominal region accompanied by mucocutaneous pallor, diaphoresis, dizziness, weak pulses, and hypotension. Physical examination revealed peripheral cyanosis, tachypnoea, tachycardia, and decreased breath sounds in the left hemithorax. His previous history included an uncomplicated 23 mm KD incidentally discovered by an Angio-CT, hypertension, pulmonary emphysema, and hypertriglyceridemia. A chest X-ray was performed, showing a left pleural effusion. A presumptive diagnosis of rKA was established. An Angio-CT showed a 62 mm aneurysmatic diameter from the apex to the opposite wall of the aorta and a mural thrombus occupying 70% of its lumen without evidence of an intimal flap.

This patient underwent an emergent FET. During surgery, a rKA of approximately 70 × 70 mm was found, which caused a left haemothorax. Dissection of the rKA was performed, and antegrade cerebral perfusion was continuously given through both carotid arteries. FET technique was conducted, the end of the arch graft was inserted distally, the Dacron tube was then anastomosed to the descending aorta's origin, and a retrograde stent-graft was deployed in the descending aorta. The surgery required a 54-min cardiac arrest and a 70-min clamp period.

On the first postoperative day, the patient presented a right pneumothorax, requiring a closed thoracostomy. Later, he presented a left pneumothorax, hypoxic-ischemic encephalopathy, multiple runs of atrial fibrillation, and disseminated intravascular coagulation, complicated with a gram-negative septic shock. The patient died on the 11th postoperative day due to cardiorespiratory arrest secondary to sepsis.

### Case number 2 (FET + ascending aortic reconstruction)

A 69-year-old male was referred to our institution for six months of interscapular severe pain associated with dyspnoea. An extra-institutional Angio-CT showed a fusiform dilation of the aortic root and a type 1 KA. The patient presented a headache at the examination, and a hypertensive crisis was diagnosed. Beta-blockers were initiated due to a high risk of acute aortic syndrome. An institutional Angio-CT revealed dilation of the ascending aorta of 45 mm, an aortic knob of 42 mm, and a type 1 rKA of 59 mm from the apex to the opposite wall of the aorta without evidence of an intimal flap.

The patient underwent an ascending aortic reconstruction + FET. Dissection of the ascending aorta, the aortic arch, including the neck vessels, as well as the first portion of the descending aorta, was conducted. A terminal-lateral anastomosis of the left carotid artery to a Dacron tube of 8 mm was performed. An antegrade endovascular straight stent-graft of 167 × 40 mm was deployed, and the distal part of the ascending aorta and aortic arch was replaced with a Dacron graft of 34 mm. A zone III distal anastomosis was created between the stent-graft and the Dacron. The supra-aortic branches were reimplanted as a single tissue patch with a termino-lateral anastomosis to the Dacron tube of 34 mm. Aortic clamp time lasted 179 min, circulatory arrest 52 min, and extracorporeal circulation 275 min.

**Fig. 1:**
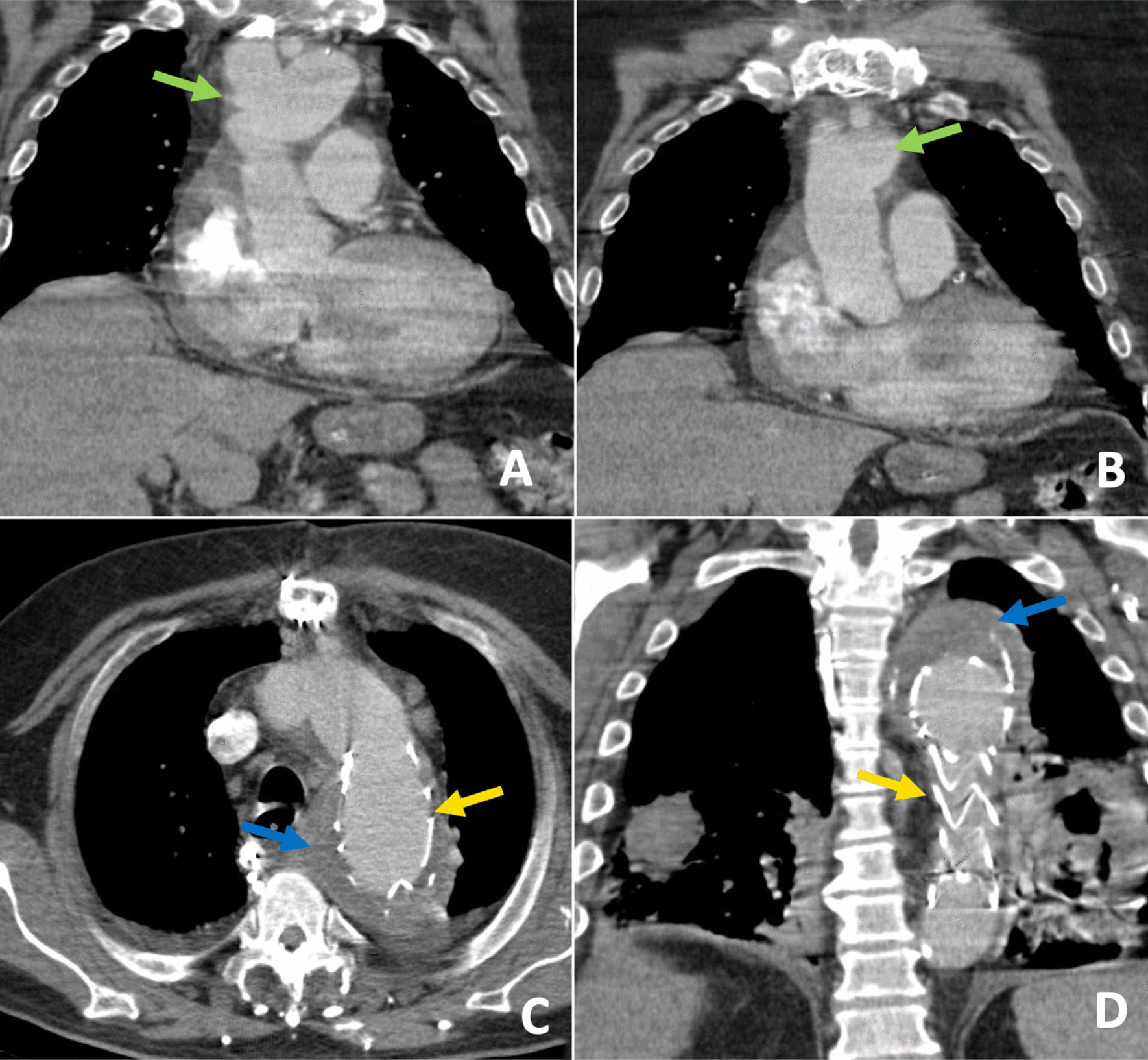
Case 1, non-gated postoperative chest CT at five years follow-up. **A**, **B**, and **D** Coronal images show appropriate positioning and patency of the thoracic graft with no further dilation or other complications (yellow arrows on **C** and **D**). A correct re-implantation of the supra-aortic trunks is also visualised (green arrows on **A** and **B**). A thrombosed aneurysmal sac is also evidenced (blue arrows on **C** and **D**). **D** Note the predominantly bi-basal opacities in the context of community-acquired pneumonia

The patient presented hyperactive delirium and postoperative mediastinitis, which were successfully treated. After full recovery, the patient was discharged after ten days with no further complications. Five years later, the patient was admitted due to respiratory symptoms. A CT scan showed pneumonia, the KA was excluded, and a successful arch reconstruction was visible (Fig. 1).

### Case number 3 (supra-aortic debranching + TEVAR)

A 58-year-old male was referred to our institution with an extra-institutional chest X-Ray that revealed a left pleural effusion and mediastinal enlargement and an Angio-CT that showed a rKA of 70 mm from the apex to the opposite wall of the aorta. The patient had a previous history of hypertension. He presented to the ER with a sudden onset of intense oppressive chest pain radiating to the left arm, accompanied by severe shortness of breath and general malaise. Physical examination revealed a hypotensive, tachycardic, and tachypnoeic patient, and initial arterial blood gases showed hyperlactatemia.

**Fig. 2:**
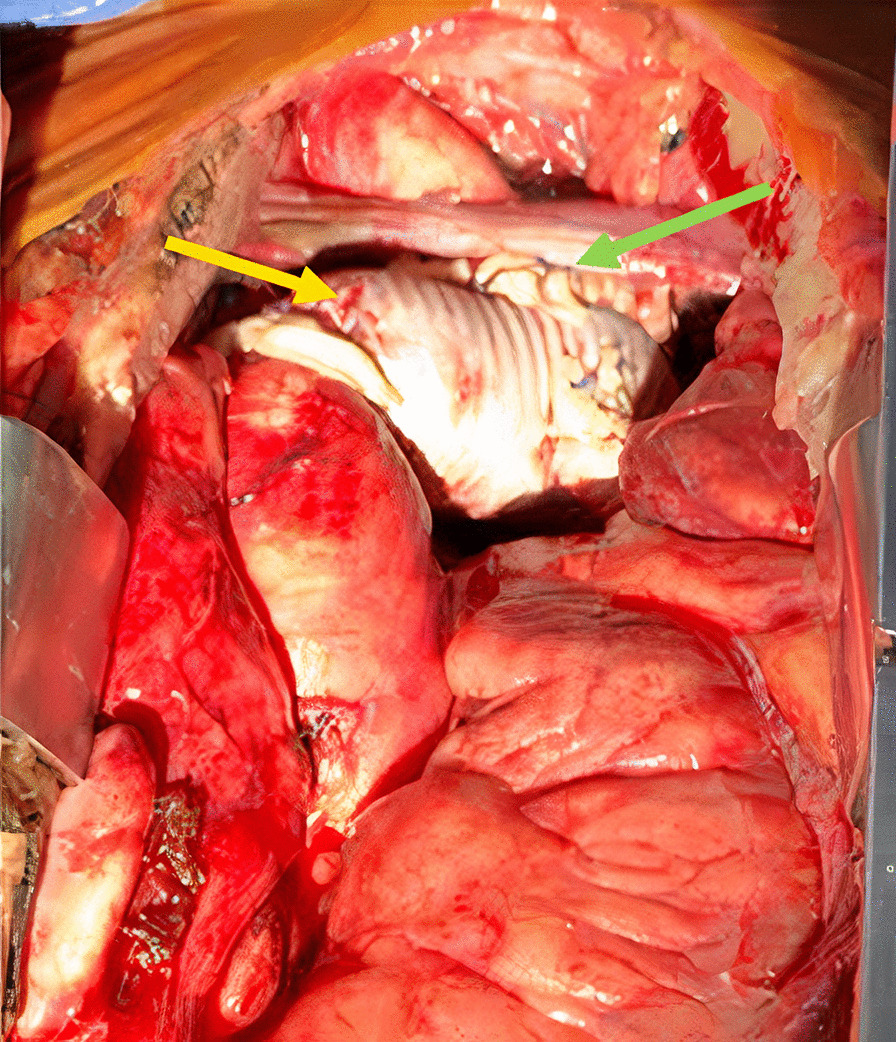
Intraoperative image of the rKA open stage of the hybrid repair. The complete aortic arch replacement with no acute bleeding and correct positioning is shown (yellow arrow). The reconstruction and reimplantation of the supra-aortic vessels can be observed (green arrow)

The patient immediately underwent surgery for supra-aortic debranching + TEVAR. Selective anterograde cerebral perfusion was given through the left carotid artery. A retrograde stent-graft was deployed in zone-2. A KA of approximately 60 × 80 mm was dissected. Subsequently, the neck vessels were anastomosed to a 24 mm Dacron tube. An 8 mm Dacron was anastomosed to the aLScA and proximally to the ascending aorta (Fig. 2). After 171 min, the aortic clamp was removed, and the cardiac arrest was reversed after 48 min.

**Fig. 3:**
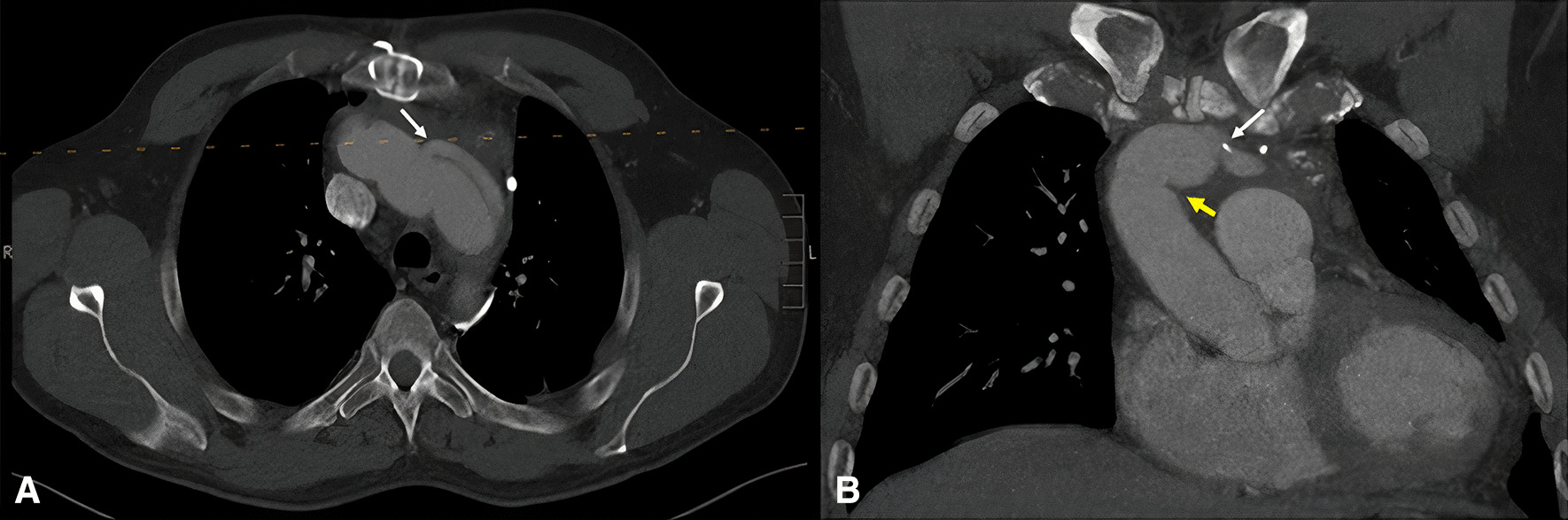
**A** Axial and **B** coronal CT angiography images showing the anastomosis site of the reimplanted left subclavian artery and the aortic arch (white arrow) as well as the anastomosis between the distal ascending aorta and the Dacron tube (yellow arrow)

The patient presented multiple complications during hospitalisation, including a vasoplegic hypovolemic shock, delirium, bacteraemia, and drop foot, all of which were appropriately managed without any long-term consequences. The patient was discharged 20 days after admission without any sequelae. After two years of follow-up and an Angio-CT showed an adequate evolution (Fig. 3).

## Discussion and conclusions

KD's management has different approaches depending on the patient's comorbidities, clinical presentation, anatomy, and the surgeon's expertise and personal preferences [1, 2, 23]. Some authors recommend surgical correction only in symptomatic patients since this population has been found to have an accelerated growth rate and a higher risk of rupture [1, 2]. It is also proposed to intervene in all patients with a KD diameter > 30 mm at the base of the KD or a distance from the apex of the KD to the opposite wall of the aorta of > 50 mm [1, 8]. 

After three years of follow-up, elective open and endovascular approaches demonstrated similar cumulative mortality; 16 and 18.2%, respectively. In this context, endovascular approaches may be an attractive alternative as they are associated with a lower incidence of postoperative pneumonia, shorter duration of invasive mechanical ventilation and shorter hospital-stay length. [2, 10, 24–27]. Evidence regarding hybrid approaches such as FET or thoracic endovascular aortic repair (TEVAR) plus supra-aortic debranching has been increasing recently, showing similar results in the short term compared to the endovascular management [18, 19, 23–26, 28, 29].

A systematic review of the literature compared the three approaches for KD. All are relatively safe and effective, showing no differences in outcomes, including 30-day mortality or stroke. The only significant difference was in the group of patients that underwent endovascular interventions, showing a higher number of re-interventions, mainly due to the appearance of endoleaks (11.6%) [30]. Each approach will be discussed in more detail in the following paragraphs, and their advantages and disadvantages are summarised in Table 3 [23, 27, 28, 30–36].

**Table 3:** Summary of the advantages and disadvantages of the different approaches and techniques for the correction of KD and KA

**Reported surgical approaches for KD and KA**					
Technique		Advantages		Disadvantages	
		General	Specific	General	Specific
**Open surgical approaches**					
Thoracotomy		Complete treatment Suitable for difficult anatomy Symptomatic relieve Secure brain perfusion Treatment of associated congenital abnormalities		Extensive surgical incisions Multiple access strategies required Not suitable for patients with high surgical risk Requirement of general anesthesia	
Sternotomy					
Supraclavicular					
**Total endovascular approaches**					
TEVAR	Isolated	Minimally invasive procedure Suitable for patients with high surgical risk Lower incidence of pneumonia Lower duration of mechanical ventilation Lower hospital length of stay		Not symptomatic relive in patients with complete vascular ring Size reduction is not always achieved High rates of endoleaks and stent-graft migration High rates of re-intervention Arterial-esophageal fistula	Arm claudication Spinal ischemia predisposition Vertebrobasilar insufficiency
	Oclussion of the distal tract of the AScA		Prevents retrograde blood flow Decompression of the aneurysmal sac		
	Chimney + Periscope		Complete treatment Improved proximal sealing Maintain patency of the AScA		Gutter-related endoleak Requirement of surgeon-modified or custom-made thoracic endografts
**Hybrid approaches**					
TEVAR + Supra-aortic debranching	Carotid-to-subclavian bypass	Complete treatment Avoid thoracotomy Possible use of late TEVAR Treatment of associated congenital abnormalities	1 or 2 staged procedure Suitable for patients with high surgical risk	Requirement of sternotomy Requirement of general anesthesia	Branches occlusion Type I and Type III endoleaks Stent-graft migration
	Carotid-to-carotid bypass				
	Carotid-to-subclavian transposition				
Frozen elephant trunk			1-staged procedure Suitable for difficult anatomy Secure blood thigh fixation proximally Avoid Type I and Type III endoleaks Avoid stent-graft migration Symptomatic relieve		Espinal cord injury Extended cardiac arrest time
Elephant trunk			2-staged procedure for complete treatment Similar to FET		High early-mortality rates Aortic ruptures between procedures

Given its low incidence, the evidence regarding the management of rKA needs more robust and extensive studies reporting long-term outcomes. There is even lower evidence about the most appropriate surgical approach for type-1 non-dissecting rKA. As a result, in the following section, we will describe the existing data about the different surgical approaches for all types of rKA.

### Hybrid approach

Hybrid approaches are generally preferred as they are less morbid, avoid thoracotomy, and permit a posterior TEVAR if necessary. [28, 31]. Several techniques have been described. The most commonly performed are the FET and TEVAR + Supra-aortic debranching [30]. 

The TEVAR + supra-aortic debranching technique offers the possibility of performing a primary repair or alternative correction (embolization) of the AScA [31]. However, it does not solve the problem of the proximal landing zone and has been associated with complications such as branch occlusion, endoleaks, and stent-graft migration [31, 33]. One of its main advantages is that it can be performed in one or two stages, depending on the patient's surgical risk and general clinical condition [20]. 

The FET technique solves the issue of a limited proximal landing zone by performing a blood thigh proximal fixation and, therefore, decreases the risk of Type I and Type III endoleaks or stent-graft migration. This is achieved by approaching the arch through a sternotomy and suturing the stent-graft directly into the aortic arch. In addition, this approach allows direct access to the heart and the ligamentum arteriosum, which is highly useful when approaching patients with complex anatomies [28, 33]. It also decreases the potential risk of aortic rupture between procedures associated with the traditional elephant trunk, as it is a 1-staged procedure. [28, 32, 34]. The main disadvantage is that it requires longer cardiac arrest times, increasing the risk of cardiac, brain, and visceral ischemia [32, 33]. Likewise, it has been described that if the distal stent-graft is deployed below T7-T8 vertebral levels, the risk of spinal cord ischemia increases [32]. 

Ben Abdallah et al. [15] presented a 74-year-old male patient with a 81 mm contained rKA, who underwent a one-stage hybrid procedure. Initially, a TEVAR was performed associated with cervical debranching of the aLSA; then, a left carotid endarterectomy. This decision was related to the risk of haemorrhagic stroke due to cardiac arrest. Although the primary procedure was successful, the patient underwent two secondary procedures, one for vertebrobasilar insufficiency and the other for a right cervical lymphocele. After a four-month follow-up, the patient had fully recovered.

Another case report by Singh et al. [17] reported a 62‐year‐old African American male patient with acute onset tearing chest pain. An angio-CT identified a dissecting 40 mm Type I rKA with an intramural hematoma. The patient underwent a total arch replacement consisting of a right carotid-subclavian bypass, followed by open debranching of the bilateral carotid arteries and a zone II arch replacement with proximal intrathoracic ligation of aRSA and LSCA. Posteriorly, the patient underwent a FET. A retrograde TEVAR due to a type of IA endoleak was conducted in a second intervention. The patient presented a right lacunar infarct requiring a tracheostomy and percutaneous gastrostomy. After discharge, the patient persisted with mild residual left‐sided weakness.

Sica et al. [20] reported the case of a 74-year-old male patient with a saccular 70 mm Type II rKA associated with an anomalous course of the left brachiocephalic vein, which coursed posteriorly to the ascending aorta, and joined the right brachiocephalic vein to form the superior vena cava. Besides, a hematoma of the descending aorta was found, forming an extrapleural hematoma. The patient underwent a TEVAR, and a vascular plug was placed into the first segment of the aLSA. Twenty hours later, in a second surgical time, a left carotid-axillary bypass was performed. The length of hospital stay was 15 days. The patient presented transient tetraplegia. Nevertheless, at a 2-year follow-up, there were no residual or additional complications.

The evidence described above is related to our cases. We consider this approach promising for rKA as it allows an individualized approach for each patient. Furthermore, it is feasible in emergent situations as it is an effective, less invasive alternative compared to open approaches and does not require extensive planning or more specific resources like total endovascular approaches.

### Open approaches

Historically, this has been the mainstay approach for rKA, as shown in a review by Cinà et al. [8]. Besides providing good access to the affected structures, it allows symptomatic relief and treatment of any related congenital or acquired cardiac abnormalities. It is particularly valuable for patients with complex anatomical variations as it allows complete visualization of the heart and great vessels, and in patients at high risk of stroke as it provides the possibility of securing brain perfusion. [23, 30, 36]. Nevertheless, it is highly invasive and morbid, requiring extensive surgical incision and multiple accesses strategies.

A study by Ikeno et al. [2] presented two patients with rKA who underwent emergent open surgery. The first patient included was a 77-year-old male with a dissecting rKA of 50.9 mm in diameter, who underwent an extensive aortic repair with selective anterograde cerebral perfusion. The authors reported no long-term postoperative complications in this patient. The second patient included was an 89-year-old male patient that presented a non-dissecting rKA with a diameter of 52 mm and underwent a total aortic arch replacement with selective antegrade cerebral perfusion. In-hospital mortality secondary to pneumonia was reported.

A case report by Kaki et al. [11] described a 73-year-old female patient with a 35 mm Type II rKA. An initial open proximal anastomosis was performed with a branched vascular graft. Then, the vascular graft was clamped, the aLSA was reconstructed, and reperfusion of the upper body was started through the rKA distal anastomosis. The patient presented pneumonia, which was treated adequately. The total hospital stay length was 70 days.

### Endovascular approach

The evidence regarding the utility of this approach for rKA is null. This may be due to the requirement of extensive surgical planning since, for the complete treatment of the pathology, it is necessary to use chimney and periscope techniques that require surgeon-modified and custom-made thoracic endografts. Additionally, favourable anatomy with no evidence of anomalous supra-aortic branches or alterations of the descending aorta and a healthy landing zone with no calcifications, dilatations, or thrombi is required.

However, in centres with adequate experience and resources available, a total endovascular approach could be considered for select emergent cases, such as patients with contained rKA or those at high risk of surgical complications., However, its major role is in preventing rupture or dissection of KA. A proximal anterograde chimney technique with a retrograde approach to improving the proximal seal and a concomitant periscope technique should be performed to maintain the AScA patency [35, 36].

TEVAR with or without embolization of the AScA have also been described, but with complications such as arm claudication, predisposition to spinal ischemia, subclavian steal syndrome, and vertebrobasilar insufficiency [35]. Vascular plugs or coil embolization can prevent retrograde blood flow, and sometimes it has been related to decompression of the aneurysmal sac [31]. Additionally, given the sharply-curved distal arch (small radius curvature) when a KA is present, there is a predisposition to kinking, collapse of the thoracic endograft, or aortic wall injury by stent fractures [31, 35]; In this setting, some authors suggest the use of bare metal stents to decrease the risk of mechanical complications associated with stent fracture [36]. These techniques may be suitable in patients with a Type III KA.

It should be noted that endovascular techniques are contraindicated in the presence of compressive symptoms as the failure of clinical symptoms to improve has been described in the setting of complete vascular rings or if aneurysmal size reduction is not successful [23, 28, 30, 34]. 

In conclusion, technique selection must be individualised for each patient, depending on various factors such as the availability of resources, the patient's status (symptoms, hemodynamic condition, surgical risk, comorbidities), the different anatomical variations that may be present, and the surgeon's ability and preference. In our experience, FET can be a feasible procedure for patients with rKA requiring emergent surgical repair. However, other hybrid or open procedures could also be successfully performed. More robust studies are required to assess each approach's quality and long-term results to establish the most effective and safe for rKA.

## Data Availability

The data are not available for public access due to patient privacy concerns but may be available from the corresponding author upon reasonable request.

## Abbreviations

KD: Kommerell’s diverticulum
KA: Kommerell’s aneurysm
rKA: Ruptured Kommerell’s aneurysm
AScA: Aberrant subclavian artery
aRScA: Aberrant right subclavian artery
aLScA: Aberr: Aberrantt left subclavian artery
LScA: Left subclavian artery
RScA: Right subclavian artery
Angio-CT: Computed tomography angiogram
CT: Computed tomography
FET: Frozen elephant trunk
TEVAR: Thoracic endovascular aortic repair
ER: Emergency room
VATS: Video-assisted thoracoscopic surgical
